# Food Insecurity Reduces the Chance of Following a Nutrient-Dense Dietary Pattern by Brazilian Adults: Insights from a Nationwide Cross-Sectional Survey

**DOI:** 10.3390/nu14102126

**Published:** 2022-05-19

**Authors:** Michelle Alessandra de Castro, Mariane de Mello Fontanelli, Carlos Alberto Nogueira-de-Almeida, Mauro Fisberg

**Affiliations:** 1 School Feeding Coordination, São Paulo City Hall, Sao Paulo 01069-900, Brazil; 2 School of Arts, Sciences and Humanities, University of São Paulo, Sao Paulo 03828-000, Brazil; marianefontanelli@usp.br; 3 Medical Department, Federal University of São Carlos, Sao Carlos 13565-905, Brazil; dr.nogueira@me.com; 4 Instituto Pensi, Fundação José Luiz Egydio Setubal, Hospital Infantil Sabará, Sao Paulo 01227-200, Brazil; mauro.fisberg@gmail.com

**Keywords:** food insecurity, dietary patterns, adults, diet surveys, cross-sectional analyses, Brazil

## Abstract

Dietary patterns derived by data-driven techniques are still scarce in the food security context and may be a useful tool to inform policymakers to promote adequate and healthy diets for vulnerable populations. We investigated the association between food security status and dietary patterns among 28,127 Brazilian adults using data from the 2017–2018 Household Budget Survey. Food security status was measured by the Brazilian Food Insecurity Scale. Food items reported in two 24 h dietary recalls were combined into food groups. Dietary patterns were derived from exploratory factor analysis, and participants were grouped according to their factor scores by K-means cluster analysis. Multiple logistic regression models were fitted to investigate the association between food security status and clusters of adherence to dietary patterns. Four dietary patterns were derived: ‘Brazilian breakfast style’ (cluster 3), ‘Brazilian Traditional staple foods’ (cluster 2), ‘Beverages, ready-to-eat and convenience foods’ (cluster 4), and ‘Fruits, vegetables, and whole grains’ (cluster 1). After adjustments, food insecurity was inversely associated with adherence to the ‘Fruits vegetables, and whole grains’ pattern (OR = 0.75, 95% CI: 0.63–0.89). Findings suggest food insecurity negatively impact the adoption of a nutrient-dense dietary pattern and highlight the critical role of policy actions in a scenario of increasing food insecurity.

## 1. Introduction

Food security has been defined as access to sufficient, safe, and nutritious food to meet one’s dietary needs and food preferences for a healthy life [1]. Food availability, accessibility, utilization, and stability in food access are built-in aspects of this concept, and food insecurity may prevail when one of these pillars is lacking. As a result, quality and quantity of food consumed may be affected, and the possibility of hunger experience comes into play [2]. Since 2014, food insecurity has been increasing worldwide, and the prevalence of severe or moderate food insecurity was estimated to be 25.9% in 2019, reaching 2 billion people globally [3]. These numbers are even worse considering the potential impact of the COVID-19 pandemic, suggesting that the current efforts are not enough to end hunger, achieve food security, and improve nutrition (Sustainable Development Goals 2 Targets 2.1 and 2.2) [3,4]. Importantly, food insecurity is negatively linked to health outcomes, with a potential impact in the double burden of malnutrition, i.e., the coexistence of undernutrition and diet-related noncommunicable diseases, which affect most low- and middle-income countries [5,6].

The way people modify their diets when facing food insecurity has been previously studied and seems to vary according to the country income level and cultural particularities [3]. In general, results have shown that dietary quality tends to be lower in food-insecure than food-secure populations, and a decrease in nutritious foods such as fruits, vegetables, meat and dairy products have been described, as well as a lower intake of vitamins A, C, B6, and calcium, iron, magnesium, and zinc [7,8]. Historically, investigations linking food insecurity and dietary outcomes looked into individual nutrients, foods, or food groups, and also used hypothesis-driven dietary patterns, such as the Health Eating Index, to investigate the association between dietary patterns and food security status [8,9,10]. However, dietary patterns derived by data-driven approaches are still scarce in the food security context and may provide a way to explore existing patterns and eating behaviors in the population, accounting for cultural variations and the complex combinations of foods [11,12,13]. It might also be a useful method to locate vulnerable population subgroups within general population dietary patterns.

The aim of the current investigation was to explore the association between dietary patterns and food security status among Brazilian adults in a nationwide sample before COVID-19 pandemic. The policy implications of this investigation arise from the escalation of moderate and severe food insecurity in South America, from 18.8% in 2014 to 28.5% in 2019 [3]. Additionally, recent data from Brazil suggest that around 55% to 60% of the population is facing food insecurity in the context of COVID-19 pandemic [14,15]. Therefore, a better understanding about dietary patterns according to food security status of Brazilian adults may be useful to inform policymakers from similar food-exporting developing countries the opportunities to improve access to nutritious diets by vulnerable populations with the aim of preventing the recurrence of this scenario in the future.

## 2. Materials and Methods

### 2.1. Study Population

Data were used from the National Dietary Survey and the Household Budget Survey (HBS), both conducted from July 2017 to July 2018 by the Brazilian Institute of Geography and Statistics (IBGE, *Instituto Brasileiro de Geografia e Estatística*), the official agency of Brazilian Population Statistics. The surveys were designed to collect data on consumption expenditure, life conditions, as well as nutritional information in a representative sample of Brazilians. Participants were randomly selected via two-stage cluster sampling: census tracts (primary sampling unit) and households (secondary sampling unit). Census tracts were stratified according to geographical (region, urban/rural areas, and administrative division) and household income [16]. A total of 5504 census tracts were selected, and 57,920 households had sociodemographic, expenditure, and life conditions information collected during a nine-day in-home interview. A subsample of 20,112 households (~35% of the total sample), including 46,164 participants aged >10 years (y), also completed at least one 24 h dietary recall (24HR) [17]. The present study included a total of 28,153 adults (20–59 y, both sexes, non-pregnant, non-lactating) with sociodemographic, life condition and dietary data collected. The association between food security status and dietary patterns was performed in 28,127 adults with complete information.

This investigation was performed in accordance with the Brazilian Law #5534 from 14 November 1968, which guarantees confidentiality of the information collected by all national census. All ethical principles laid down in the Declaration of Helsinki and in the Brazilian Resolution Number 196/96 on research involving human subjects were followed.

### 2.2. Food Security Data

Food security status was measured by the Brazilian Food Insecurity Scale (EBIA, *Escala Brasileira de Insegurança Alimentar*), an adapted scale from that proposed by the United States Department of Agriculture (USDA) and validated to the Brazilian population [18,19]. The scale was answered by the household reference person, which was selected using the following criteria: (1) the member responsible for housing expenses, (2) indicated by others household members, or (3) the oldest person in the household, and information was generalized to all household members [18]. EBIA is a psychometric scale that assesses food security status during the past three months using yes/no questions, including 14 items for households with members aged <18 years and 8 items for households without members aged <18 years. The higher number of affirmative responses indicates greater food insecurity. The final score is categorized as food security (score 0 or no affirmative responses), mild food insecurity (1–5 points for households with members aged <18 years, and 1–3 points for households without members aged <18 years), moderate food insecurity (6–9 and 4–5 points for households with members aged <18 years and households without members aged <18 years, respectively), and severe food insecurity (10–14 and 6–8 points, respectively). For analytical purposes, the final score of EBIA was further categorized as food security and food insecurity (i.e., mild, moderate, or severe food insecurity).

### 2.3. Dietary Data Collection

Individual dietary data were collected using two non-consecutive 24HRthroughout all days of the week and seasons of the year. Both 24HR were collected by face-to-face interviews at participant’s homes following procedures described in the USDA Automated Multiple Pass Method [20].

Individuals were advised by interviewers to inform the amounts of foods, culinary preparations and beverages consumed (including water) in household measures as well as to name eating occasions and report their clock time, place of consumption, cooking methods, and added seasonings. In households, interviewers entered the 24HR data into a specific mobile computing software developed by IBGE to collect dietary data. This mobile software converted household measures of foods and beverages into standard units of weight or volume (i.e., grams or milliliters) automatically. The software database encompassed 1832 options of foods and allowed the registration of food items added to beverages or foods before consumption, such as table sugar, artificial sweeteners, honey, butter/margarine, olive oil, etc. Quality control of the 24 h was conducted during and after interview aiming to identify and correct misreporting in real-time.

Energy and nutrient content of each food item reported in 24 h was obtained by the Brazilian Food Composition Table (TBCA-USP), version 7.0, developed by the Food Research Center (FoRC) at University of São Paulo (USP), available at http://www.fcf.usp.br/tbca (accessed on 10 December 2021), in accordance with standards and guidelines for the generation, compilation and use of food composition data of FAO/INFOODS (Food and Agriculture Organization/International Network of Food Data System).

### 2.4. Foods Grouping

A total of 1508 different foods were reported in both 24HR and then collapsed into forty-seven food groups. In the first step, foods were combined according to the similarity of the nutrient profile [21] (e.g., all types of brewed tea were combined into the ‘tea’ group) and the particular dietary habits and culinary usage of the Brazilian population [22] (e.g., ‘beans’ group included brown and black beans that are usually eaten in South and Southeast regions of Brazil, while ‘cowpea beans’ group are usually eaten in North and Northeast regions). In the second step, food groups were analyzed by the correlation matrix [23]. Similar food groups with positive and significant correlations were aggregated into a single food group (e.g., leafy and non-leafy vegetables were grouped; beef and pork meat were grouped into ‘red meat’), and similar food groups with negative and significant correlations were maintained in different groups (e.g., soybeans, lentils, chickpeas and snow peas were maintained separated in the ‘other legumes’ group owing to negative correlation with ‘beans’ and ‘cowpea beans’ groups). A detailed description of food groups composition is provided in Supplementary Table S1.

Food group intakes (in grams) were adjusted for within-person variation through the web based statistical modeling technique Multiple Source Method (MSM), version 1.0.1, updated in 2020. The MSM was developed within the European Food Consumption and Validation Project as a suitable technique for estimating the usual nutrient and food intakes (including those episodically consumed) based on two or more short-term dietary methods per individual such as the 24HR [23,24]. The effects of day of the week (weekday vs. weekend) and atypical day of dietary intake (no vs. yes) were considered as adjustments in the models.

### 2.5. Covariates

The following sociodemographic variables related to household members were considered: age group (20–29 years, 30–39 years, 40–49 years, and 50–59 years), sex (male, female), education level (0–4, 5–9, 10–12, and ≥13 years of study), and ethnicity (‘white or yellow’ and ‘black, brown or indigenous’). Self-reported ethnicity categorization was based on Brazilian law #12711 from 29 August 2012, which provides admission into public universities and institutions.

Lifestyle data included: body mass index (BMI), whether the participant followed a specific diet (yes/no), food variety score (FVS, tertiles), number of eating occasions (1–3, 4–6, and ≥7), number of main meals (1, 2, and 3), and subjective evaluation of the family’s standard of living regarding diet (good, satisfactory, and bad). BMI was estimated based on self-reported weight and height information and was classified as underweight (BMI < 18.5 kg/m^2^), healthy weight (BMI 18.5–24.9 kg/m^2^), overweight (BMI 25–29.9 kg/m^2^), and obese (BMI ≥ 30 kg/m^2^) [25]. FVS was defined as the number of unique food items consumed in the first 24HR [26,27]. An eating occasion was defined as each episode participants reported the consumption of foods or beverages [17]. Eating occasions reported as ‘breakfast’, ‘lunch’, and ‘dinner’ were considered main meals. The subjective evaluation of the standard of living in relation to diet was assessed by asking the household reference person “how do you evaluate your family’s standard of living in relation to diet?” [18].

Characteristics related to the household were also considered: area (urban or rural), region of the country (North, Northeast, Southeast, South, and Midwest), per capita family income (≤1 minimum wage, MW ; >1 and ≤3 MW, and >3 MW), number of household members (≤ 3, 4–6, and ≥7), children <5 y in the household (yes/no), individuals >60 y in the household (yes/no), sex of the household reference person (male, female), age of the household reference person (≤39 y, 40–59 y, ≥60 y), ethnicity of the household reference person (‘white or yellow’, ‘black, brown or indigenous’), and education level of the household reference person (0–4, 5–9, 10–12, and ≥13 years of study). Per capita family income was estimated by summing all monetary and non-monetary income reported by family members divided by the number of family members, and minimum wage was 954.00 Brazilian Real (BRL) in 2018 (equivalent to USD 298.53, 1 USD = 3.1957 BRL on 15 January 2018).

### 2.6. Statistical Analysis

Descriptive statistics (percentages and 95% Confidence Intervals—95% CI) of socioeconomic, demographic, anthropometric and lifestyle characteristics of population were estimated according to the food security status (secure vs. insecure).

Dietary patterns were derived from exploratory factor analysis (EFA) with principal component extraction method (PCF) and Varimax orthogonal rotation. The input variables were the usual amounts of food group intakes, in grams. The sample adequacy for EFA was verified by the Kaiser–Meyer–Olkin test (KMO test = 0.52) and Bartlett’s sphericity test (*p*-value < 0.001). KMO values > 0.50 and Bartlett’s sphericity test <0.05 were considered acceptable for EFA analysis [28]. Minimum eigenvalues of 1.5 and Cattell’s scree test (plot of eigenvalues) were analyzed as initial criteria to identify the number of factors to be retained for interpretation. In Cattell’s scree test, the number of factors until the first inflection point of the curve were considered a criterion for interpretation (Supplementary Figure S1), as performed in previous dietary patterns studies [29,30]. Food groups with positive factor loadings can be interpreted as contributing directly to the factor, whereas food groups with negative factor loadings can be interpreted to be inversely correlated with the factor [23]. For interpretation and labeling purposes, food groups with factor loadings ≥|0.20| were considered significant.

After factor extraction, factor scores were predicted for each individual by least squares regression method. Regression factor scores predict the location of each individual on each factor [31], i.e., allow the estimation of individual’s adherence to the factors. Individuals were grouped according to their factor scores into mutually exclusive groups by K-means cluster analysis using Euclidian distance. The number of clusters was defined to be equal to the number of factors extracted. K-means cluster analysis creates non-hierarchical groups of individuals according to the means or centroids of the input variables (factor scores) and maximizes the distance between clusters [32]. Hence, individuals with high similarity in a factor score but with high dissimilarity to the other factor scores were grouped into the same cluster. The mean factor scores and the percentage of individuals according to food security status were estimated in each cluster. Large positive means of a factor score in a cluster were considered for identifying the groups of individuals with adherence to that factor.

Multiple logistic regression models were fitted to investigate the association between food security status (dependent variable; 0—food security; 1—food insecurity) and clusters of adherence to dietary patterns (independent variables). Covariates were considered based on the association between each covariate and food security status (*p*-value < 0.20) in univariate analysis, and a stepwise forward procedure was adopted for the inclusion of covariates in the multiple models. Covariates were retained in the final model if they were associated with food security status or adjusted other variables more than |10%|. Three models were presented, and variables were included in the following order: model (1) univariate analysis; model (2) model 1 + lifestyle variables (subjective evaluation of the family’s standard of living in relation to diet, FVS, whether the participant followed a specific diet); model (3) model 2 + sociodemographic characteristics related to the household (per capita family income, region, number of people in the household, education level, ethnicity, sex, and age of the household reference person). The models included sociodemographic variables related to the household instead of household members based on the strength of association and literature background [8,33]. Goodness-of-fit test for survey-weighted logistic regression models was used to assess model fitness (*p*-value > 0.05).

All statistical analyses were conducted in Stata 12.0^®^ software. Descriptive statistics and regression models considered the complex sampling design of the National Dietary Survey 2017–2018 (svy family commands). Differences in sociodemographic, economic, anthropometric, and lifestyle variables as well as in adherence to dietary patterns according to food security status were evaluated through Pearson’s Chi-square test with the Rao and Scott second-order correction. Two-sided *p*-values < 0.05 were considered as significant.

## 3. Results

Characteristics of Brazilian adult population according to food security status are presented in Table 1. Overall, the prevalence of food insecurity status was 40.5% (mild food insecurity: 27.1%, moderate food insecurity: 8.8%, and severe food insecurity: 4.6%) and was higher among women, those who self-reported black, brown, or indigenous ethnicity, that had until 4 y of study, and was lower among those aged 50 to 59 years. Regarding lifestyle characteristics, the prevalence of food insecurity was higher among those in the first of FVS’s tertile, who had 1 to 3 meals a day, and who evaluated the family’s standard of living in relation to diet as bad. A higher proportion of the population facing food insecurity was also seen in the rural area, North region, and those with per capita family income ≤1 MW, living in households with ≥7 members, and with children aged <5 years, among those whose reference person were female, black, brown, or indigenous ethnicity, and individuals with the reference person with lower education level.

**Figure 1:**
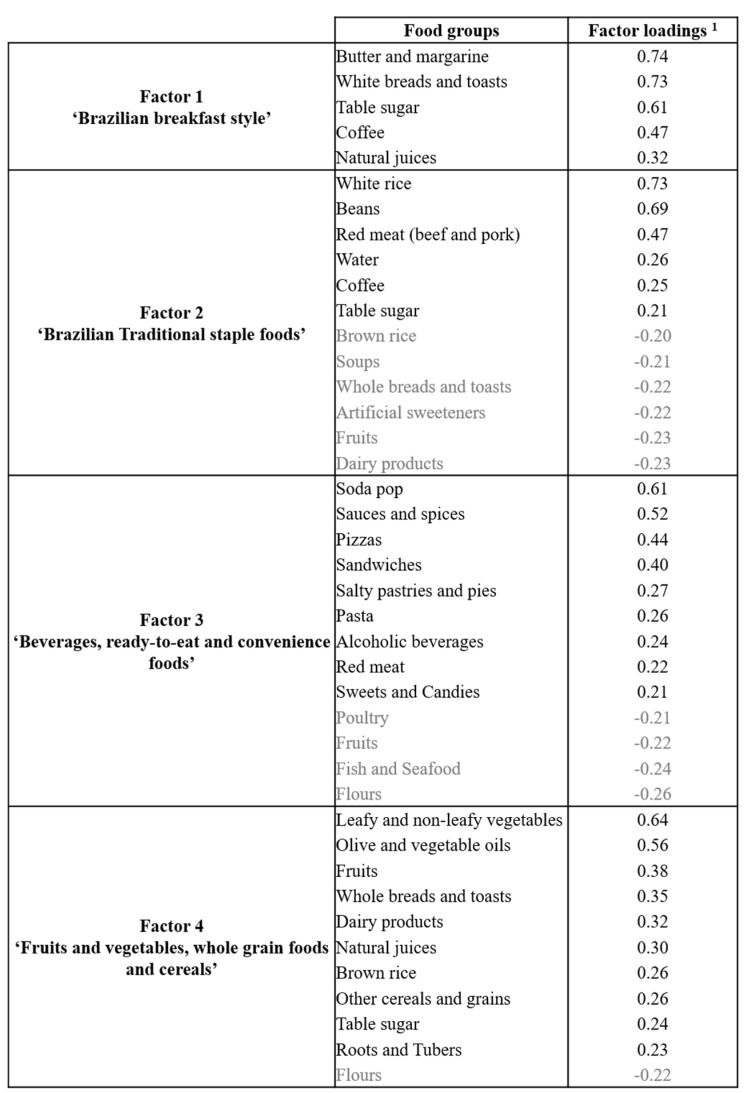
Composition of Brazilian adults’ dietary patterns derived by exploratory factor analysis, Household Budget Survey 2017–2018, Brazil. ^1^ Presented according to factor loadings’ decreasing order. Only food groups with factor loadings ≥|0.20| were presented with the purpose to facilitate interpretation of patterns. Negative factor loadings are highlighted in grey.

A total of four dietary patterns were derived which accounted for 16.7% of total variance of intake (Figure 1). The first dietary pattern, labeled ‘Brazilian breakfast style’, had higher and positive loadings on butter and margarine, white breads and toasts, and table sugar followed by coffee and natural juices. The second dietary pattern, labeled ‘Brazilian traditional staple foods’, had higher and positive loadings on white rice and beans groups, followed by red meat, water, coffee, and table sugar. Negative loadings were observed for brown rice, soups, whole breads and toasts, artificial sweeteners, fruits, and dairy products. The third dietary pattern, labeled ‘Beverages, ready-to-eat and convenience foods’, had higher and positive loadings on soda pop, sauces and spices, pizzas, and sandwiches, followed by salty pastries and pies, pasta, alcoholic beverages, red meat, and sweets and candies. Negative loadings were observed for poultry, fruits, fish and seafood, and flours. The fourth dietary pattern, labeled ‘Fruits, vegetables, and whole grains’, had higher and positive loadings on leafy and non-leafy vegetables, and olive and vegetable oils followed by fruits, whole-grain breads and toasts, dairy products, natural juices, brown rice, other grains and cereals, table sugar, and roots and tubers. Negative loading was observed for the flours group.

The clusters of adherence to the dietary patterns comprised individuals with the largest positive mean scores for each of the derived dietary patterns. The first cluster included 4098 individuals (14.6% of the sample) with adherence to the ‘Fruits, vegetables, and whole grains’ pattern. The second cluster was the largest, comprising 13,346 individuals (47.4% of the sample) with adherence to the ‘Brazilian traditional staple foods’ pattern. The third cluster included 6050 individuals (21.5% of the sample) with adherence to the ‘Brazilian breakfast style’ pattern, and the fourth cluster comprised 4659 individuals (16.5% of the sample) with adherence to the ‘Beverages, ready-to-eat and convenience foods’ pattern (Table 2). Sociodemographic characteristics of adults according to clusters of adherence are presented in Supplementary Table S2.

The percentage of individuals in clusters of adherence to the dietary patterns differed according to food security status (Table 3). The two most prevalent dietary patterns among food-insecure individuals were ‘Brazilian traditional staple foods’ and ‘Brazilian breakfast style’, which were prevalent in 75% of these individuals. In turn, the two most prevalent dietary patterns among food-secure individuals were ‘Brazilian traditional staple foods’ and ‘Beverages, ready-to-eat and convenience foods’ patterns, which were prevalent in about 58% of these individuals. ‘Brazilian traditional staple foods’ pattern was more prevalent among food insecure individuals relative to food secure ones (49.8% vs. 35.1%). Differently, the adherence to the ‘Fruits, vegetables, and whole grains’ pattern was larger among food-secure individuals than food-insecure ones (21.3% vs. 9.6%).

Clusters of adherence to dietary patterns were also differently associated with food security status even after adjustment for independent variables (Table 4). In the univariate analyses (model 1), participants in the clusters of adherence to ‘Fruits, vegetables, and whole grains’, ‘Brazilian breakfast style’, and ‘Beverages, ready-to-eat and convenience foods’ dietary patterns presented lower odds to be facing food insecurity compared to participant’s adherent to the ‘Brazilian traditional staple foods’ dietary pattern (odds ratio (OR) = 0.32 95% CI: 0.27–0.37; OR = 0.86, 95% CI: 0.77–0.96; OR = 0.47, 95% CI: 0.41–0.55, respectively). After adjusting for potential confounders related to lifestyle characteristics (model 2), the cluster of adherence to the ‘Brazilian breakfast style’ was no longer associated with food security status (OR = 0.93, 95% CI: 0.82–1.05). In the final model, further adjusted for sociodemographic characteristics related to the household (model 3), participants in the cluster of adherence to the ‘Fruits, vegetables, and whole grains’ dietary pattern presented 25% lower odds to be in food insecurity compared to those in the ‘Brazilian traditional staple foods’ dietary pattern (OR = 0.75, 95% CI: 0.63–0.89). The cluster of adherence to the ‘Brazilian breakfast style’ and ‘Beverages, ready-to-eat and convenience foods’ were no longer associated with food security status in the final model (OR = 0.98, 95% CI: 0.86–1.11; OR = 0.93, 95% CI: 0.79–1.09, respectively).

## 4. Discussion

To the best of our knowledge, this was the first nationally representative study that investigated the associations between dietary patterns and food security status among Brazilian adults. Four dietary patterns were derived using data-driven techniques: ‘Brazilian breakfast style’, ‘Brazilian Traditional staple foods’, ‘Beverages, ready-to-eat and convenience foods’, and ‘Fruits, vegetables, and whole grains’; however, only the subgroup of the population following the dietary pattern mainly composed of healthier foods (foods that are recommended by major national and international dietary guidelines) [34], i.e., ‘Fruits, vegetables, and whole grains’ had lower odds of facing food insecurity after adjusting for potential confounders. Results indicate that food insecurity negatively impact the guarantee of available, regular, and permanent access to foods that are related to the protection of malnutrition in all its forms and highlight the critical role of policy actions in this issue. Nevertheless, less healthy dietary patterns, such as ‘Beverages, ready-to-eat and convenience foods’, were also identified among individuals classified as food secure by the EBIA, suggesting that other factors related to food choices and preferences as well as lifestyle characteristics may affect the adoption of dietary patterns among individuals.

Between 2017 and 2018, 40.5% of the Brazilian adult population faced mild, moderate, or severe food insecurity. This alarming scenario points to the reverse of gradual improvements seen from 2004 to 2013 in the country, when food insecurity affected ~35% and ~23% of Brazilian’s households, respectively [35,36]. Characteristics associated with food security status identified in this study are highly consistent with those in the literature [8,33]. Higher proportions of the Brazilian population facing food insecurity were seen in rural areas, North and Northeast regions, among those with lower levels of education and income, in households with a higher number of members, and whose household reference person were female and/or black, brown, or indigenous [8,33].

With regard to the dietary pattern analysis, the first pattern identified (‘Brazilian breakfast style’) was the second most prevalent among food-insecure adults (25.3%) and was characterized by white breads and toasts, butter and margarine, table sugar, coffee, and natural juice. These foods are typically consumed at a breakfast meal among Brazilian adults, as observed by Santos et al. [37], who identified the ‘Traditional’ breakfast pattern with similar food composition in adults living in São Paulo. The ‘Brazilian breakfast style’ pattern may be indicative of a low to moderate nutritional quality meal according to the Brazilian Breakfast Quality Index (BQI) [38], considering that coffee without milk, white breads, and butter and margarine were in top five most frequently consumed foods by individuals with low to medium BQI scores as identified by the authors [38].

The second pattern, labeled ‘Brazilian Traditional staple foods’, was characterized by traditional foodstuffs namely rice and beans, followed by red meat, water, coffee, and table sugar. Similar dietary patterns were previously identified in Brazilian cross-sectional studies [30,37,39]. An expected finding was the high adherence to the ‘Brazilian Traditional staple foods’ pattern among food-insecure individuals (49.8%), considering the large participation (i.e., higher factor loadings) of rice and beans in this pattern. These traditional foodstuffs for Brazilians play an important role by providing nutrition, especially among food insecurity scenarios. Rice and beans are two staple and cultural foods widely consumed in Brazil, especially by economic vulnerable groups that are more prone to food insecurity [10], and are important sources of energy and nutrients such as carbohydrates, vegetable protein, dietary fiber, potassium, and B vitamins [40,41], but are poor in other micronutrients such as vitamins A, C, D, and E. Evidence towards more frequent or regular consumption of beans among adults and older adults living in food-insecure households was previously identified by de Araújo et al. [10] in a cross-sectional study with users of a primary healthcare service in Belo Horizonte, Brazil. It should be pointed out that this dietary pattern is still incomplete in terms of staple foods composition, considering that the Brazilian staple foods basket is composed of rice, beans, meat, coffee, sugar, milk, flour, potato, fruits (banana), non-leafy vegetables (tomato), bread, oil, and butter [42].

The third pattern, labeled ‘Beverages, ready-to-eat and convenience foods’, was composed of soda pop, sauces and spices, pizzas, sandwiches, salty pastries and pies, pasta, alcoholic beverages, red meat, and sweets and candies. It resembled the ‘Western’, ‘Modern’, ‘Convenience’, and ‘Processed foods’ dietary patterns that are composed primarily of foods of low nutritional quality (i.e., energy dense, high-fat, high-salt, high-sugar, and low-fiber) and markers of unhealthy diet identified in national [30,37,39] and international [43,44] studies, such as soda pop, sauces and spices, pizzas, sandwiches, salty pastries and pies, pasta, alcoholic beverages, red meat, and sweets and candies. This pattern was more prevalent among food-secure individuals (22.8%) than food-insecure ones (15.3%). No differences were observed among food-secure and food-insecure individuals evaluated by de Araújo et al. [10] for similar low nutritional quality foods, i.e., fried foods, meats and processed meats, salty snacks, cookies, sweets, and sugar-sweetened beverages. It is worth mentioning the widespread consumption of sugar in Brazil, such as added sugar in soda pop and sweets and candies in the ‘Beverages, ready-to-eat and convenience foods’ dietary pattern, but also as table sugar in ‘Brazilian breakfast style’, ‘Brazilian Traditional staple foods’, and ‘Fruits, vegetables, and whole grains’ dietary patterns. A similar scenario was seen in other countries [43,44,45].

The fourth dietary pattern, labeled ‘Fruits, vegetables, and whole grains’, was quite similar to the ‘Prudent’, ‘Healthy’, and ‘Health-conscious’ dietary patterns [43,44,46] and was characterized by leafy and non-leafy vegetables, olive and vegetable oils, fruits, whole breads and toasts, dairy products, natural juices, brown rice, other grains and cereals, table sugar, and roots and tubers. This pattern was composed of foods of higher nutritional quality (i.e., low-energy and nutrient-dense foods) that are markers of a healthy diet. This pattern was more prevalent among food-secure individuals (21.3%) than food-insecure ones (9.8%). The adherence to this pattern was associated with a 25% lower odds of food insecurity in comparison to the ‘Brazilian Traditional staple foods’ pattern, after adjusting for sociodemographic and lifestyle characteristics. Therefore, it is possible that for Brazilians under food insecurity is more affordable and economical to follow the traditional pattern by purchasing energy-dense and less perishable foods, such as rice and beans, rather than nutrient-dense and more perishable foods, especially fruits and vegetables, identified in the ‘Fruits, vegetables, and whole grains’ dietary pattern.

The low consumption of healthier and nutritious foods was previously documented among food-insecure populations and seems to be affected by cultural aspects and country income level [3,10,47]. In Kenya and Sudan, lower-middle-income countries, people facing moderate food insecurity decreased the consumption of most food groups, but increased staples in their diet [3]. In Mexico and Samoa, upper-middle-income countries, foods that are commonly cheaper on an energy basis, such as cereals, roots, tubers and plantains, were more consumed by people facing moderate food insecurity, while more expensive foods (meat and dairy) were less consumed when compared with those who are food secure. As the severity of food insecurity increased, a decrease in fruit and dairy consumption were also observed in Mexico [3]. We were not able to assess dietary pattern according to food insecurity severity, but considering the high inequality rates in Brazil, different scenarios may coexist in the country [48].

Taken together, the results suggest that food insecurity impairs the adoption of a higher nutrient content dietary pattern and depicts access to healthier foods as one probable cause of diet-related disparities, also contributing to a higher burden of noncommunicable diseases among food insecure subgroups of the population [3,6]. Nevertheless, the adoption of a nutritious dietary pattern was not uniform among the food-secure subgroup of the population, and multiple factors such as taste, preparation time, distance to food markets, and cost may influence the adopted patterns [49,50]. Therefore, food-secure subgroups may have more opportunities to decide on the adoption of a healthier diet, despite its higher cost [3].

The findings of this study have a number of policy implications that must be considered. First, the price is a strong determinant of food consumption and may be one reason for disparities in diet quality among food-secure and food-insecure individuals [3,51]. The majority of foods included in the ‘Fruits and vegetables, whole grain foods and cereals’ pattern cost more per energy provided, and projections suggest increasing trends in prices of fresh foods [52,53]. However, despite costing more on an energy basis, fruits, vegetables, and dairy offer higher nutrient content (protein, fiber, vitamins A, C, D, and E, calcium and iron) per Brazilian Real spent [53]. Importantly, data on the price of whole grain foods are still scarce in Brazil, but evidence points to the higher price of these foods when compared to refined grain options [54,55]. In this sense, a better understanding of local and global cost drivers and the implementation of policy actions to provide access to healthy foods for vulnerable populations are crucial to tackle the widening diet-disease gap between high- and low-income subgroups.

It should be pointed out that, since the COVID-19 pandemic outbreak, income losses and supply chains disruptions led to a 14% rise in food prices and a reduced amount of money to buy foods [48], which contributed to a sharp increase in global food insecurity in 2020, especially among poor populations. In Brazil, food basket items had a significant price increase during this time—soy oil (103.8%), rice (76.0%), tomatoes (52.76%), fruits (25.40%), and meat (17.97%)—and food insecurity affected more than half of the Brazilian population, with 19.1 million people experiencing hunger [14,15]. A detrimental impact on diet quality was also observed, particularly reductions in meat, fruits, and vegetables, which affected low-income subgroups to a greater extent [14].

Second, beyond financial access, physical barriers also affect food security status. Inadequate road infrastructure, a lack of public/private transportation, and long distances to reach food markets are common in low-income neighborhoods, and food deserts (low availability of food retailers) and food swamps (higher availability of less healthy food retailers than healthful food retailers) can be a local reality [3,56,57]. Multilayered interventions aiming at improving the environment infrastructure (e.g., transportation and roads), investments in storage and processing techniques that preserve nutrient content of fresh foods (e.g., cool storage systems and cold chains), incentives for healthful food retailers/increase in healthier food options in already existing stores, and bringing family farmers closer to consumers offer opportunities to increase access to healthier foods in these localities [3,56].

Third, national food assistance programs in Brazil, such as the National School Meal Program [58] and Worker’s Food Program [59], are pathways to guarantee access to nutritious foods, and efforts to keep them functioning during this challenging time are needed. Cash transfer programs also contributed to avert food scarcity, but in general, incentives had small value, short-term duration, and limited scope (not covering everyone in need), reinforcing the importance of building permanent social protection systems [48]. Recently, Healthy Food Prescription Programs are emerging as a potential public health tool by providing participants (most often those experiencing food insecurity or cardiometabolic conditions) subsidized or free healthy food items [60]. Finally, it is important that these actions take place in a food system under transformation, where a change of perspective from food quantity to food quality is required to address food insecurity and malnutrition in a sustainable manner [3,61].

This study has several strengths, including the large and nationally representative sample of Brazilian adults, the quality control in data collection, the application of a validated scale of food security measurement for Brazilian population (EBIA), the adjustment for the within-person variation of short-term dietary intake data, and the originality of the analysis conducted (i.e., principal factor analysis combined with cluster analysis to dietary patterns investigation in food security context). However, some limitations deserve to be acknowledged. First, the cross-sectional design does not allow inferring the causal relationships between food security and dietary patterns, owing to the chance of reverse causality. Second, both food security and dietary intakes were estimated through self-reported information, therefore it is important to consider the chance of measurement error, memory bias and misreporting of energy intake [62].

Finally, food security status was obtained by interviewing the household reference person and this information represent their self-perceived dimension about the risk of lacking foods at home or the unavailability of sufficient foods for family consumption. Moreover, the household food security status was used to estimate the individual food security status enabling the association with individual dietary patterns. For this, all family members living in the same household and sharing the same family income were classified according to their household food security status. It worth mentioning that EBIA measures food access related to income, and it does not consider food items cultivated for a family’s own consumption, collective cultivation practices, and food exchange, which could contribute to food security, especially in rural areas [63].

## 5. Conclusions

Food insecurity among Brazilian adults threatens the adoption of a healthy and nutrient-dense diet. The dietary pattern composed of fruits and vegetables, whole grains, and cereals was inversely associated with food insecurity status when compared to a diet mainly composed of traditional staple foods such as rice and beans. It would be ideal for both health and the preservation of cultural habits of the population, and the improvement of the Brazilian Traditional dietary pattern in order to include fruits, vegetables and whole grains, especially among socially vulnerable groups. For this, ensuring permanent access to healthy, sustainable, and affordable foods by regulating food prices, expanding access of vulnerable groups to food and nutrition programs, reducing social inequities, promoting education and food sovereignty actions combined with health and nutrition surveillance and the assessment of the effectiveness of those policies must be the major goals of policymakers from developing countries.

## Figures and Tables

**Table 1:** Characteristics of the Brazilian adult population (*n* = 28,153) according to food security status based on the Household Budget Survey 2017–2018, Brazil.

Characteristics	Food Security			Food Insecurity ^1^			*p*-Value ^2^
	*n*	% ^3^	95% CI	*n*	%	95% CI	
Total	15,878	59.52	(58.13–60.89)	12,275	40.48	(39.11–41.87)	-
Characteristics of the household members							
Age group, years							
20–29	3544	57.94	(55.65–60.2)	3121	42.06	(39.8–44.35)	<0.001
30–39	4193	57.44	(55.21–59.63)	3405	42.56	(40.37–44.79)	
40–49	4147	59.7	(57.57–61.8)	3127	40.3	(38.2–42.43)	
50–59	3994	63.52	(61.4–65.59)	2622	36.48	(34.41–38.6)	
Sex							
Male	7635	60.41	(58.91–61.9)	5703	39.59	(38.1–41.09)	0.004
Female	8243	58.63	(57.11–60.14)	6572	41.37	(39.86–42.89)	
Ethnicity							
White and yellow	7119	70.09	(68.25–71.86)	3377	29.91	(28.14–31.75)	<0.001
Black, brown and indigenous	8747	51.45	(49.8–53.09)	8889	48.55	(46.91–50.2)	
Education level, years							
0–4	1693	41.66	(38.88–44.48)	2450	58.34	(55.52–61.12)	<0.001
5–9	4406	52.26	(50.32–54.18)	4153	47.74	(45.82–49.68)	
10–12	5744	59.1	(57.19–60.98)	4277	40.9	(39.02–42.81)	
≥13	4035	78.48	(76.27–80.53)	1395	21.52	(19.47–23.73)	
Lifestyle characteristics							
Body mass index							
Underweight	316	53.95	(46.44–61.29)	288	46.05	(38.71–53.56)	0.055
Healthy weight	6723	58.78	(57.02–60.53)	5349	41.22	(39.47–42.98)	
Overweight	6204	61	(59.19–62.78)	4521	39	(37.22–40.81)	
Obese	2635	58.68	(56.23–61.09)	2117	41.32	(38.91–43.77)	
Followed a specific diet							
Yes	2183	61.42	(58.98–63.79)	1595	38.58	(36.21–41.02)	0.092
No	13,695	59.23	(57.76–60.7)	10,680	40.77	(39.3–42.24)	
Food variety score (FVS)							
Tertile 1 (2–10 food items)	6125	51.68	(49.69–53.68)	6314	48.32	(46.32–50.31)	<0.001
Tertile 2 (11–12 food items)	4279	60.74	(58.7–62.75)	3235	39.26	(37.25–41.3)	
Tertile 3 (13 food items)	5474	69.26	(67.29–71.17)	2726	30.74	(28.83–32.71)	
Number of meals							
1–3	1482	54.24	(49.97–58.44)	1306	45.76	(41.56–50.03)	0.010
4–6	9958	60.18	(58.56–61.77)	7644	39.82	(38.23–41.44)	
≥7	4438	60.11	(58.03–62.16)	3325	39.89	(37.84–41.97)	
Main meals							
3	12,986	59.46	(58.01–60.88)	10,073	40.54	(39.12–41.99)	0.115
2	2665	60.78	(58.26–63.24)	1996	39.22	(36.76–41.74)	
≤1	227	50.59	(39.71–61.42)	206	49.41	(38.58–60.29)	
Evaluation of the standard of living in relation to diet							
Good	11,374	72.01	(70.37–73.6)	5004	27.99	(26.4–29.63)	<0.001
Satisfactory	4331	45.17	(42.99–47.37)	5880	54.83	(52.63–57.01)	
Bad	173	12.92	(9.83–16.78)	1391	87.08	(83.22–90.17)	
Household characteristics							
Area							
Urban	12,530	61.19	(59.64–62.73)	9333	38.81	(37.27–40.36)	<0.001
Rural	3348	48.97	(46.39–51.56)	2942	51.03	(48.44–53.61)	
Region							
North	1692	37.96	(34.18–41.89)	2440	62.04	(58.11–65.82)	<0.001
Northeast	4517	46.59	(44.72–48.46)	5200	53.41	(51.54–55.28)	
Southeast	4531	64.98	(62.28–67.6)	2498	35.02	(32.4–37.72)	
South	2859	76.88	(74.12–79.41)	840	23.12	(20.59–25.88)	
Midwest	2279	63.3	(59.88–66.59)	1297	36.7	(33.41–40.12)	
Family income per capita ^4^							
≤1 minimum wage	5133	39.49	(37.59–41.42)	8085	60.51	(58.58–62.41)	<0.001
>1 and ≤3 minimum wages	7883	67.35	(65.3–69.33)	3779	32.65	(30.67–34.7)	
>3 minimum wages	2862	89.17	(86.82–91.14)	411	10.83	(8.86–13.18)	
Number of household members							
≤3 members	9231	66.17	(64.59–67.71)	5533	33.83	(32.29–35.41)	<0.001
4 to 6 members	6159	54.18	(51.88–56.45)	5771	45.82	(43.55–48.12)	
≥7 members	488	31.09	(25.66–37.1)	971	68.91	(62.9–74.34)	
Children <5 years							
Yes	2707	48.96	(45.93–51.99)	2961	51.04	(48.01–54.07)	<0.001
No	13,171	62.15	(60.63–63.64)	9314	37.85	(36.36–39.37)	
Individuals > 60 years							
Yes	2986	60.04	(57.2–62.82)	2185	39.96	(37.18–42.8)	0.6883
No	12,892	59.41	(57.88–60.92)	10,090	40.59	(39.08–42.12)	
Sex of the household reference person							
Male	10452	63.31	(61.62–64.98)	6933	36.69	(35.02–38.38)	<0.001
Female	5426	52.72	(50.61–54.81)	5342	47.28	(45.19–49.39)	
Age of the household reference person, years							
≤39	4884	57.49	(55.03–59.9)	4129	42.51	(40.1–44.97)	0.0694
40 to 59	9050	60.62	(58.82–62.39)	6686	39.38	(37.61–41.18)	
≥60	1944	60.3	(57.01–63.49)	1460	39.7	(36.51–42.99)	
Ethnicity of the household reference person							
White and yellow	7004	69.97	(67.84–72.03)	3273	30.03	(27.97–32.16)	<0.001
Black, brown and indigenous	8855	51.73	(49.98–53.48)	8995	48.27	(46.52–50.02)	
Education level of the household reference person, years							
0–4	2453	43.65	(41.03–46.31)	3383	56.35	(53.69–58.97)	<0.001
5–9	5060	54	(51.59–56.4)	4445	46	(43.6–48.41)	
10–12	5054	61.78	(59.37–64.13)	3336	38.22	(35.87–40.63)	
≥13	3311	78.81	(76.05–81.33)	1111	21.19	(18.67–23.95)	

^1^ Comprises the categories of mild food insecurity, moderate food insecurity and severe food insecurity of the Brazilian Food Insecurity Scale (EBIA); ^2^ Obtained from the Chi-square test considering the sample design of the study; ^3^ % considers the sample design of the study; ^4^ Minimum wage equivalent to BRL 954.00 in 2018.

**Table 2:** Mean factor scores per cluster and food security status among Brazilian’s adult population (*n* = 28,153), Household Budget Survey 2017–2018, Brazil.

Factor Scores	Cluster 1 (*n* = 4098)			Cluster 2 (*n* = 13,346)			Cluster 3 (*n* = 6050)			Cluster 4 (*n* = 4659)			Prob > F ^2^
	Mean	(SE)	*p*-Value ^1^	Mean	(SE)	*p*-Value	Mean	(SE)	*p*-Value	Mean	(SE)	*p*-Value	
Factor score—Factor 1	−0.32	(0.02)	-	−0.42	(0.01)	-	1.42	(0.02)	-	−0.24	(0.02)	-	<0.001
Food security	−0.34	(0.03)	0.122	−0.43	(0.01)	0.278	1.42	(0.03)	0.865	−0.24	(0.02)	0.737	<0.001
Food insecurity	−0.26	(0.04)		−0.41	(0.01)		1.41	(0.03)		−0.25	(0.03)		<0.001
Factor score—Factor 2	−0.66	(0.03)	-	0.28	(0.02)	-	0.06	(0.02)	-	−0.43	(0.02)	-	<0.001
Food security	−0.68	(0.04)	0.151	0.30	(0.02)	0.283	0.09	(0.03)	0.123	−0.46	(0.03)	0.151	<0.001
Food insecurity	−0.59	(0.05)		0.26	(0.02)		0.02	(0.03)		−0.38	(0.04)		<0.001
Factor score—Factor 3	−0.40	(0.02)	-	−0.29	(0.01)	-	−0.10	(0.01)	-	1.63	(0.03)	-	<0.001
Food security	−0.36	(0.03)	0.006	−0.21	(0.01)	<0.001	−0.04	(0.02)	<0.001	1.63	(0.03)	0.999	<0.001
Food insecurity	−0.51	(0.04)		−0.38	(0.01)		−0.17	(0.02)		1.63	(0.05)		<0.001
Factor score—Factor 4	1.66	(0.03)	-	−0.38	(0.01)	-	−0.04	(0.02)	-	−0.12	(0.02)	-	<0.001
Food security	1.71	(0.03)	0.001	−0.27	(0.01)	<0.001	0.07	(0.03)	<0.001	−0.06	(0.03)	<0.001	<0.001
Food insecurity	1.49	(0.05)		−0.49	(0.01)		−0.17	(0.02)		−0.26	(0.04)		<0.001

^1^ *p*-value: mean test of factor scores between food security status within each cluster, ^2^ Prob > F: mean test of factor scores by food security status between clusters, Factor 1—‘Brazilian breakfast style’ pattern; Factor 2—‘Brazilian Traditional staple foods’ pattern; Factor 3—‘Beverages, ready-to-eat and convenience foods’ pattern; Factor 4—‘Fruits, vegetables, whole grains and cereals’ pattern.

**Table 3:** Prevalence of Brazilian adults in the clusters of adherence to dietary patterns according to food security status (*n* = 28,153), Household Budget Survey 2017–2018, Brazil.

Food Security Status	Cluster 1 (*n* = 4098)		Cluster 2 (*n* = 13,346)		Cluster 3 (*n* = 6050)		Cluster 4 (*n* = 4659)		*p*-Value ^1^
	‘Fruits, Vegetables, and Whole Grains’ Pattern		‘Brazilian Traditional Staple Foods’ Pattern		‘Brazilian Breakfast Style’ Pattern		‘Beverages, Ready-to-Eat and Convenience Foods’ Pattern		
	%	(95% CI)	%	(95% CI)	%	(95% CI)	%	(95% CI)	
Food security	21.30	(19.93–22.74)	35.14	(33.75–36.55)	20.73	(19.57–21.94)	22.83	(21.51–24.22)	<0.001
Food insecurity	9.58	(8.67–10.57)	49.82	(48.02–51.62)	25.28	(23.80–26.82)	15.33	(13.91–16.85)	

^1^ *p*-value: Pearson’s Chi-square test corrected for the sample design by Rao–Scott’s second-order correction (1984).

**Table 4:** Logistic regression models for the association between food insecurity and the clusters of adherence to dietary patterns among Brazilian adults, Household Budget Survey 2017–2018, Brazil.

Models (*n* = 28,127) ^1^	OR	SE	95% CI	*p*-Value
**Model 1—univariate model**				
Cluster 2—‘Brazilian Traditional staple foods’ pattern	1.00 (ref.)			
Cluster 1—‘Fruits, vegetables, and whole grains’ pattern	0.32	0.02	(0.27–0.37)	<0.001
Cluster 3—‘Brazilian breakfast style’ pattern	0.86	0.05	(0.77–0.96)	0.008
Cluster 4—‘Beverages, ready-to-eat and convenience foods’ pattern	0.47	0.03	(0.41–0.55)	<0.001
**Model 2—model 1 + lifestyle variables ^2^**				
Cluster 2—‘Brazilian Traditional staple foods’ pattern	1.00 (ref.)			
Cluster 1—‘Fruits, vegetables, and whole grains’ pattern	0.43	0.03	(0.36–0.5)	<0.001
Cluster 3—‘Brazilian breakfast style’ pattern	0.93	0.06	(0.83–1.06)	0.227
Cluster 4—‘Beverages, ready-to-eat and convenience foods’ pattern	0.56	0.04	(0.48–0.66)	<0.001
**Model 3—model 2 + sociodemographic variables ^3^**				
Cluster 2—‘Brazilian Traditional staple foods’ pattern	1.00 (ref.)			
Cluster 1—‘Fruits, vegetables, and whole grains’ pattern	0.75	0.06	(0.64–0.89)	0.001
Cluster 3—‘Brazilian breakfast style’ pattern	0.98	0.06	(0.86–1.11)	0.723
Cluster 4—‘Beverages, ready-to-eat and convenience foods’ pattern	0.93	0.08	(0.79–1.09)	0.370

^1^ Only participants with complete data were included in this analysis.; ^2^ Model 2—adjusted for: subjective assessment of food, diet variety score, follow a specific diet; ^3^ Model 3—adjusted for variables in Model 2 and family income per capita, region, number of people in the household, and education level, ethnicity, sex, and age of the household reference person reference.

## Data Availability

Data used in the present study is made public available by the Brazilian Institute of Geography and Statistics (https://www.ibge.gov.br/estatisticas/sociais/saude/24786-pesquisa-de-orcamentos-familiares-2.html?=&t=microdados, 6 March 2022). The code used in this study is available upon request.

## Supplementary Materials

The following supporting information can be downloaded at: https://www.mdpi.com/article/10.3390/nu14102126/s1, Figure S1: Scree plot of eigenvalues from the exploratory factor analysis (EFA) with principal component extraction method (PCF) and Varimax orthogonal rotation; Table S1: Description of the forty-seven food groups composition used to derive dietary patterns based on the Household Budget Survey, 2017–2018, Brazil; Table S2: Characteristics of the Brazilian adult population (*n* = 28,153) according to clusters of adherence to dietary patterns, Household Budget Survey 2017–2018, Brazil.
