# Supplementary material for: Food Insecurity Reduces the Chance of Following a Nutrient-Dense Dietary Pattern by Brazilian Adults: Insights from a Nationwide Cross-Sectional Survey
Source: Nutrients. 2022 May 19;14(10):2126. doi: 10.3390/nu14102126 (PMC9143026; doi:10.3390/nu14102126)

Nutrients – Supplementary Material

Food insecurity reduces the chance of following a nutrient-dense dietary pattern by Brazilian adults: insights from a nationwide cross-sectional survey

Michelle Alessandra de Castro, Mariane de Mello Fontanelli, Carlos Alberto Nogueira-de-Almeida, Mauro Fisberg

Contents

Table S1. Description of the forty-seven food groups composition used to derive dietary patterns based on the Household Budget Survey, 2017-2018, Brazil. 2

Table S2. Characteristics of the Brazilian adult population (n=28,153) according to clusters of adherence to dietary patterns, Household Budget Survey 2017-2018, Brazil. 4

Figure S1. Scree plot of eigenvalues from the exploratory factor analysis (EFA) with principal component extraction method (PCF) and Varimax orthogonal rotation. 7

Table S1. Description of the forty-seven food groups composition used to derive dietary patterns based on the Household Budget Survey, 2017-2018, Brazil.

**Food groups**	**Food items**
White rice	Cooked white rice
Beans	Brown beans, black beans, pinto beans
Red meat	Steak beef, ground beef, beef ribs, pork chop, pork ribs, pork loin (all cooking methods)
Water	Water tap, bottled water, mineral water, sparkling water
Coffee	Brew coffee, instant coffee, cappuccino, decaffeinated coffee, espresso coffee
Table sugar	White sugar, demerara sugar, brown sugar, honey, molasses
Leafy and non-leafy vegetables	Lettuce, kale, escarole, spinach, cabbage, watercress, chard, arugula, tomato, carrot, beet, chayote, cucumber, eggplant, okra, pumpkin, zucchini
Corn	Boiled corn, grits, sweet corn mush, hominy
Cake (without fillings and toppings)	White cake, chocolate cake, cornmeal cake, orange cake, carrot cake, sponge cake (regular, diet, light)
Cowpea beans	Cooked/boiled cowpea beans
Sausages and Cold cuts	Sausages, frankfurters, ham, mortadella, salami, roast beef
Organs meats	Liver, intestine, kidney, stomach (pork, beef and poultry)
Industrialized and Sweetened beverages	Industrialized fruit juices, sugar cane beverage, energetic drinks, isotonic drinks
Poultry	Chicken, turkey, duck, mallard, goose (all cooking methods)
Alcoholic beverages	Beer, wine, spirits, cognac, champagne, vodka, liquor
Tea	Herbal tea (brewed or instant)
Olive and vegetable oils	Extra virgin olive oil, virgin olive oil, soybean oil, cottonseed oil, sunflower oil, canola oil, corn oil
Canned vegetables	Olives, canned peas, canned corn, sauerkraut, canned hearts of palm, mushrooms
Natural juices	Natural fruit juices, beet juice, carrot juice, kale juice, mixed juice of fruits and vegetables
Roots and tubers	Potato, sweet potatoes, cassava, yam (all cooking methods)
Other legumes	Cooked soybeans, lentils, chickpeas, snow peas
Bacon and Jerky beef	Bacon, jerky beef, salted meat
Cookies and Crackers	Cookies with or without fillings or toppings, crackers
Chocolate milk powder	Chocolate milk powder, regular (different brands)
Soda pop	Coke, Diet Coke, Orange soda, Lemon soda, Guaraná soda
White breads and Toasts	French bread, Italian bread, loaf bread, buns, toasts
Butter and Margarine	Butter and margarine (salted and unsalted, regular or light)
Pasta	Cooked noodles, gnocchi, lasagna, cannelloni, ravioli

Popcorn	Popcorn (sweet, salty, light)
Salty snacks	Potatoes chips, corn chips
Sweets and Candies	Cakes with fillings or toppings, sweet pies, milk candy, chocolate pudding, peanut brittle, ice cream
Salty pastries and pies	Esfiha, croquette, croissant, Italian focaccia, chicken pie, cheese pie, quiche, fried pastry
Eggs	Chicken eggs, quail eggs (all cooking methods)
Fish and Seafood	Fish, shrimps, crab, oyster, lobster, squid, octopus, canned sardine, canned tuna
Sandwiches	Hot dog, hamburger sandwich, cold cuts sandwiches, wrap, bruschetta
Flours	Corn flour, tapioca flour
Nuts and Seeds	Cashew nuts, peanut, Brazilian nuts, almonds, walnut, pecan, pistachio, flaxseeds, pumpkin seeds, sesame
Sauces and spices	Tomato sauce, white sauce, tartar sauce, mustard sauce, ketchup, mayonnaise, salad dressing, salt, vinegar
Pizza	Calabreza pizza, Pepperoni pizza, Mozzarella pizza, Margherita pizza, Vegetarian pizza
Mixed dishes (with cereals, beans, legumes, meat and/or other ingredients)	Yakisoba, Brazilian Black Beans Stew (Feijoada), rice with chicken, risotto, grits with ground beef, potato salad with tuna
Other cereals and grains	Oatmeal, oat flakes, quinoa, corn flakes, granola, wheat bran
Brown rice	Cooked brown rice, Three grain rice, Seven grain rice
Soups	Soups with or without beans, noodles, rice, corn meal and vegetables; instant soups
Whole breads and Toasts	Whole wheat bread (regular, diet or light), rye bread, whole wheat toast (regular, diet or light), rye toast
Artificial sweeteners	Artificial sweeteners (liquid or powder, not specified)
Fruits	Fresh fruits, fruit salad, coconut
Dairy products	Milk (whole, low-fat, skimmed), yogurt, cheese (white and yellow), dairy beverages, Kefir, milk smoothie

Table S2. Characteristics of the Brazilian adult population (n=28,153) according to clusters of adherence to dietary patterns, Household Budget Survey 2017-2018, Brazil.

**Characteristics**	**Cluster 1 (n=4,098)**			**Cluster 2 (n=13,346)**			**Cluster 3 (n=6,050)**			**Cluster 4 (n=4,659)**			**p-value**[b]
Characteristics of the household members	**n**	**%** [a]	**95% CI**	**n**	**%***	**95% CI**	**n**	**%***	**95% CI**	**n**	**%***	**95% CI**	
Age group, years													
20-29	694	11.99	(10.61-13.52)	2,896	37.4	(35.44-39.41)	1501	22.7	(20.97-24.52)	1574	27.91	(25.93-29.98)	<0.001
30-39	992	15.41	(14.05-16.87)	3,551	40.74	(38.78-42.73)	1735	23.31	(21.72-24.97)	1320	20.55	(18.71-22.51)	
40-49	1,133	17.55	(15.96-19.26)	3,607	43.22	(41.35-45.11)	1491	21.97	(20.47-23.55)	1043	17.26	(15.73-18.9)	
50-59	1,279	21.83	(20.16-23.6)	3,292	43.19	(41.3-45.09)	1323	22.22	(20.6-23.93)	722	12.77	(11.45-14.22)	
Sex													
Male	1,432	12.6	(11.57-13.7)	6,215	39.8	(38.41-41.21)	3300	25.62	(24.38-26.91)	2391	21.98	(20.73-23.29)	<0.001
Female	2,666	20.47	(19.34-21.66)	7,131	42.35	(40.99-43.73)	2750	19.54	(18.45-20.68)	2268	17.63	(16.54-18.77)	
Ethnicity [d]													
White and yellow	2,180	23.09	(21.44-24.83)	3,972	32.74	(31.16-34.36)	2099	20.22	(18.91-21.59)	2245	23.95	(22.49-25.47)	<0.001
Black, brown and indigenous	1,910	11.55	(10.76-12.4)	9,367	47.45	(46.04-48.85)	3947	24.36	(23.18-25.58)	2412	16.64	(15.49-17.87)	
Education level, years													
0-4	286	6.95	(5.8-8.3)	2769	64.24	(61.71-66.69)	841	22.03	(20.06-24.14)	247	6.78	(5.56-8.24)	<0.001
5-9	916	11.25	(10.04-12.57)	4529	48.62	(46.81-50.42)	1,991	25.46	(23.88-27.12)	1,123	14.67	(13.41-16.03)	
10-12	1343	14.52	(13.33-15.79)	4331	37.42	(35.79-39.07)	2,371	25.15	(23.67-26.68)	1,976	22.92	(21.39-24.52)	
≥13	1553	31.45	(29-34.01)	1717	25.78	(23.79-27.89)	847	15.04	(13.43-16.8)	1,313	27.73	(25.46-30.12)	
Lifestyle characteristics													
Body mass index [f]													
Underweight	77	13.93	(9.77-19.49)	297	42.88	(35.89-50.17)	131	24.88	(19.13-31.69)	99	18.31	(13.99-23.59)	0.412
Healthy weight	1,656	15.92	(14.74-17.18)	5805	40.85	(39.32-42.4)	2613	22.9	(21.56-24.29)	1995	20.33	(18.95-21.79)	
Overweight	1,623	16.78	(15.54-18.09)	4970	40.74	(39.09-42.41)	2370	22.68	(21.34-24.08)	1762	19.8	(18.38-21.29)	
Obese	742	17.98	(16-20.15)	2271	42.22	(39.99-44.48)	936	21.18	(19.33-23.14)	803	18.63	(16.93-20.45)	
Followed a specific diet													
Yes	1,280	38.76	(36.2-41.39)	1705	38.66	(36.22-41.16)	399	11.03	(9.64-12.58)	394	11.55	(9.82-13.54)	<0.001
No	2,818	13.21	(12.31-14.16)	11641	41.45	(40.22-42.69)	5651	24.31	(23.28-25.37)	4265	21.04	(19.97-22.15)	
Food variety score [g]													
Tertile 1 (2-10 food items)	670	6.2	(5.51-6.97)	8016	57.9	(56.07-59.72)	2193	19.49	(18.17-20.89)	1560	16.4	(14.89-18.03)	<0.001

Tertile 2 (11-12 food items)	938	14.12	(12.82-15.53)	3395	39.05	(37.17-40.96)	1957	27.29	(25.57-29.09)	1224	19.54	(17.74-21.47)	
Tertile 3 (13 food items)	2,490	32.89	(30.97-34.87)	1935	19.66	(18.42-20.96)	1900	22.76	(21.31-24.26)	1875	24.69	(23.13-26.32)	
Number of meals													
1-3	155	6.4	(4.99-8.16)	1,642	53.14	(49.07-57.18)	504	17.24	(14.74-20.06)	487	23.22	(19.53-27.38)	<0.001
4-6	2,324	15.53	(14.42-16.71)	8,291	40.5	(39.15-41.87)	3991	23.6	(22.4-24.84)	2996	20.36	(19.15-21.63)	
≥ 7	1,619	23.57	(21.95-25.27)	3,413	37.36	(35.53-39.24)	1555	22.21	(20.68-23.83)	1176	16.85	(15.42-18.39)	
Main meals													
3	3,407	16.97	(15.95-18.04)	10921	41.14	(39.9-42.39)	5283	24.15	(23.09-25.24)	3448	17.74	(16.74-18.77)	<0.001
2	663	16.04	(14.35-17.89)	2205	40.87	(38.47-43.31)	731	17.45	(15.67-19.39)	1062	25.64	(23.5-27.9)	
≤ 1	28	4.74	(2.82-7.86)	220	40.66	(31.42-50.61)	36	7.03	(4.29-11.3)	149	47.57	(36.67-58.71)	
Subjective evaluation of the standard of living - diet													
Good	2,834	19.67	(18.36-21.04)	7192	37.64	(36.15-39.16)	3307	20.94	(19.77-22.16)	3045	21.75	(20.42-23.15)	<0.001
Satisfactory	1,156	12.63	(11.33-14.05)	5205	45.38	(43.51-47.27)	2400	24.94	(23.38-26.55)	1450	17.06	(15.52-18.71)	
Bad	108	7.18	(5.29-9.68)	949	51.7	(47.59-55.79)	343	25.49	(22.01-29.31)	164	15.62	(12.17-19.83)	
Household characteristics													
Area													
Urban	3,426	17.75	(16.7-18.85)	9444	37.79	(36.54-39.05)	5011	23.04	(22-24.12)	3982	21.43	(20.3-22.6)	<0.001
Rural	672	9.02	(7.93-10.26)	3902	61.86	(59.39-64.26)	1039	19.61	(17.66-21.71)	677	9.51	(8.52-10.6)	
Region													
North	287	8.06	(6.48-9.97)	2610	57.41	(53.88-60.86)	816	23.46	(20.43-26.78)	419	11.08	(9.12-13.39)	<0.001
Northeast	1,092	10.95	(9.99-11.99)	4896	49.26	(47.54-50.99)	2528	27.02	(25.5-28.59)	1201	12.77	(11.74-13.88)	
Southeast	1,224	19.53	(17.69-21.51)	2999	36.77	(34.73-38.85)	1661	23.31	(21.58-25.15)	1145	20.39	(18.47-22.45)	
South	879	21.94	(19.99-24.03)	952	24.18	(21.8-26.73)	623	17.55	(15.76-19.49)	1245	36.33	(34-38.72)	
Midwest	616	17.97	(15.76-20.42)	1889	51.46	(48.54-54.37)	422	12.09	(10.5-13.88)	649	18.48	(16.31-20.88)	
Family income per capita [c]													
≤ 1 minimum wage	861	6.61	(5.92-7.37)	7744	54.66	(52.91-56.39)	3241	26.93	(25.47-28.44)	1372	11.8	(10.8-12.88)	<0.001
> 1 and ≤ 3 minimum wages	2,091	18.85	(17.42-20.37)	4749	35.19	(33.63-36.79)	2405	22.39	(21.03-23.81)	2417	23.56	(22.02-25.18)	
>3 minimum wages	1,146	35.69	(32.29-39.23)	853	22.62	(20.04-25.43)	404	11.87	(9.99-14.06)	870	29.82	(26.6-33.25)	
Number of household members													
≤ 3 members	2,615	19.63	(18.41-20.91)	6615	38.44	(37.1-39.79)	2926	20.75	(19.53-22.01)	2608	21.19	(19.89-22.55)	<0.001
4 to 6 members	1,398	13.88	(12.48-15.41)	5858	42.86	(40.92-44.83)	2773	24.52	(23.04-26.06)	1901	18.73	(17.16-20.41)	
≥ 7 members	85	5.25	(3.65-7.49)	873	55.61	(50.28-60.82)	351	25.85	(21.22-31.09)	150	13.29	(9.83-17.73)	

Members < 5 years													
Yes	514	10.06	(8.78-11.5)	2829	44.03	(41.53-46.57)	1363	25.17	(23.14-27.32)	962	20.74	(18.41-23.28)	<0.001
No	3,584	18.17	(17.09-19.3)	10517	40.35	(39.1-41.61)	4687	21.92	(20.89-23)	3697	19.56	(18.47-20.7)	
Members> 60 years													
Yes	799	17.3	(15.43-19.34)	2,472	40.83	(38.47-43.22)	1176	24.29	(22.23-26.48)	724	17.59	(15.64-19.71)	0.063
No	3,299	16.4	(15.4-17.45)	10,874	41.14	(39.86-42.42)	4874	22.2	(21.18-23.26)	3935	20.26	(19.16-21.42)	
Sex of the household reference person													
Male	2,503	16.62	(15.41-17.91)	8264	40.69	(39.18-42.22)	3679	22.12	(20.94-23.36)	2939	20.56	(19.23-21.96)	0.116
Female	1,595	16.43	(15.19-17.75)	5082	41.78	(40.16-43.41)	2371	23.37	(21.92-24.89)	1720	18.42	(17.11-19.81)	
Age of the household reference person, years													
≤ 39	1,082	13.78	(12.51-15.16)	4116	39.85	(37.82-41.92)	2033	22.59	(20.95-24.32)	1782	23.77	(21.78-25.89)	<0.001
40 to 59	2,519	18.32	(17.08-19.62)	7583	41.79	(40.34-43.26)	3236	22.12	(20.96-23.32)	2398	17.77	(16.63-18.98)	
≥ 60	497	16.34	(14.06-18.9)	1647	41.34	(38.61-44.13)	781	24.68	(22.28-27.25)	479	17.64	(15.35-20.19)	
Ethnicity of the household reference person													
White and yellow	2,157	23.14	(21.48-24.88)	3902	32.87	(31.09-34.69)	2029	20.04	(18.69-21.45)	2189	23.96	(22.39-25.6)	<0.001
Black, brown and indigenous	1,932	11.65	(10.77-12.6)	9434	47.18	(45.74-48.63)	4019	24.46	(23.22-25.73)	2465	16.71	(15.45-18.05)	
Education level of the household reference person, years													
0-4	435	7.48	(6.3-8.86)	3763	61.42	(59.17-63.63)	1157	21.82	(20.13-23.62)	481	9.27	(8.08-10.63)	<0.001
5-9	1109	12.07	(10.73-13.55)	4769	45.62	(43.59-47.66)	2239	25.38	(23.69-27.16)	1388	16.93	(15.53-18.43)	
10-12	1,268	16.25	(14.8-17.81)	3433	35.55	(33.59-37.55)	1990	25.03	(23.35-26.79)	1699	23.17	(21.28-25.18)	
≥13	1,286	32.56	(29.71-35.54)	1381	25.14	(22.82-27.62)	664	14.28	(12.51-16.26)	1091	28.02	(25.28-30.94)	

a % considers the sample design of the study
b Obtained from the chi-square test considering the sample design of the study
c Minimum wage equivalent to R $ 954.00 in 2018
Factor 1 - ‘Fruits and vegetables, whole grain foods and cereals’ pattern; Factor 2 - ‘Brazilian Traditional staple foods’ pattern; Factor 3 - ‘Brazilian breakfast style’ pattern; Factor 4 - ‘Beverages, ready-to-eat and convenience foods’ pattern

Figure S1. Scree plot of eigenvalues from the exploratory factor analysis (EFA) with principal component extraction method (PCF) and Varimax orthogonal rotation.

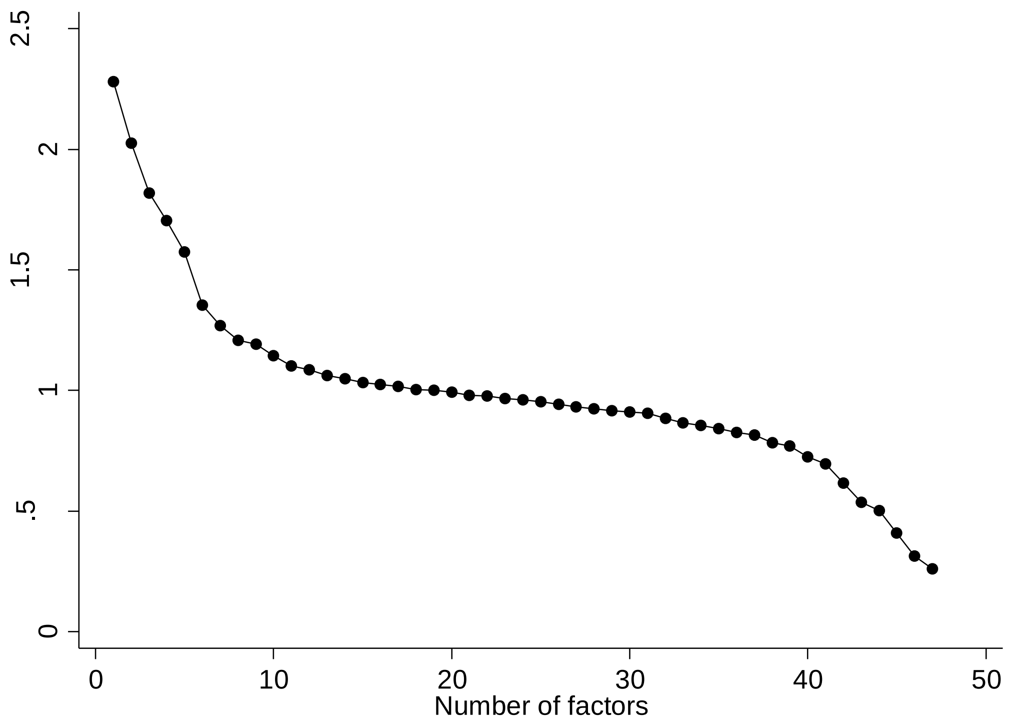

Supplement: Supplementary file 1 [file nutrients-14-02126-s001.zip › nutrients-1660918-supplementary.pdf]
